# Supplementary material for: Characterization of RNA content in individual phase-separated coacervate microdroplets
Source: Nat Commun. 2022 May 12;13:2626. doi: 10.1038/s41467-022-30158-1 (PMC9098875; doi:10.1038/s41467-022-30158-1)
Supplement: Supplementary file 1 — Supplementary Information [file 41467_2022_30158_MOESM1_ESM.pdf]

Supplementary information - Table of Contents

Supplementary Figure 1 | **Technical aspects of single-coacervate analysis** ……. 2

Supplementary Figure 2 | **Sequencing of total RNA content from coacervates** .. 3

Supplementary Figure 3 | **Effect of droplet size on consistency of the number of transcripts found in coacervates** ……………………………………………… 4

Supplementary Figure 4 | **Transcripts not detected in coacervates were not abundant in the input RNA pool** ……………………………………………… 5

Supplementary Figure 5 | **Analysis of synthetic RNA enrichment into coacervates** ……………………………………………………………… 6

Supplementary Figure 6 | **Analysis of input sensitivity**……………………………... 7

Supplementary Figure 7 | **The effect of transcript length on RNA partitioning into coacervates** ……………………………………………………………... 8

Supplementary Figure 8 | **Top motifs in enriched and randomly selected non-enriched transcripts** ………………………………………………………… 9

Supplementary Figure 9 | **Relationship between the two most abundant motifs and RNA complementarity** ………………………………………………… 10

Supplementary Figure 10 | **Sequence motif distances on transcripts** …………... 12

Supplementary Figure 11 | **Sequence motif analysis of enriched RNA transcripts derived from murine cells into coacervates** ……………………….. 13

Supplementary Figure 12 | **Motif enrichment analysis in stress granules and p-bodies** ……………………………………………………………………….. 14

Supplementary Figure 13 | **Experiment-to-experiment variation of input RNA abundances** ………………………………………………………………… 15

Supplementary Figure 14 | **Sequence motif analysis of enriched transcripts across condensate types** …………………………………………………... 16

Supplementary Figure 15 | **Global comparison of RNA content across all condensate types** ……………………………………………………………... 17

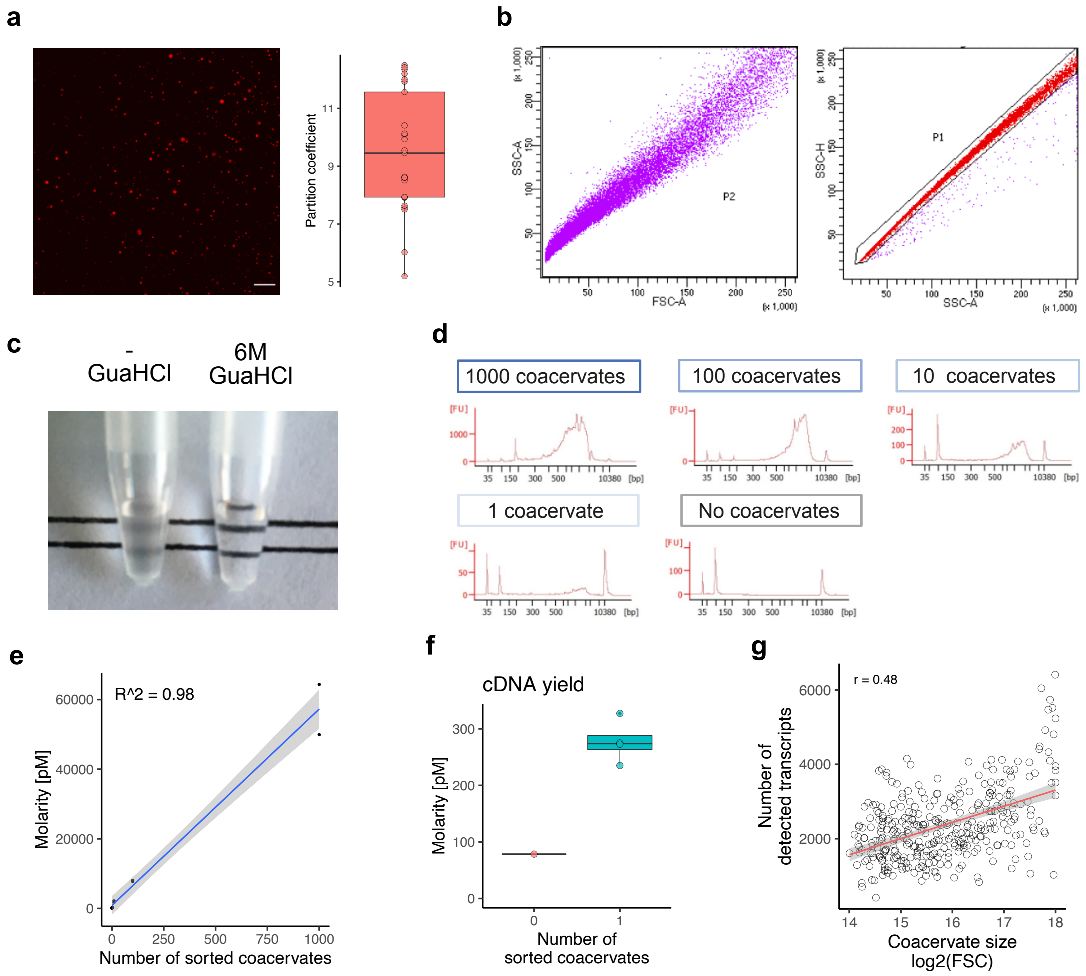

Supplementary Figure 1 | Technical aspects of single-coacervate analysis. **a**, Representative confocal microscopy picture of coacervates containing total iPSC-derived RNA stained by propidium iodide (PI). Boxplot represents the quantification of the partition coefficients measured from (n=25) displayed coacervates. Scale bar = 50µm. **b**, Representative FACS scatter plots describing coacervate size and granularity (FSC-A vs. SSC-A) and the doublet exclusion (SSC-A vs. SSC-H) gate for CM-Dex:PDDA coacervate sorting. Doublet exclusion was the only gate prior to sorting single (or bulk) coacervates into 96-well plates. **c**, Effect of 6M guanidine hydrochloride (GuaHCl) on the turbidity of CM-Dex:PDDA solution. **d**, Bioanalyzer traces for quantification and quality control of amplified cDNA prepared from multiple, single or no coacervates. **e**, Linear relationship between the number of coacervates (1000, 100, 10 1) sorted into a well and the resulting amplified cDNA library. **f**, Quantitative comparison of cDNA yield derived from libraries (n=10) prepared from either single coacervates or wells containing only primers (no coacervate sorted – negative controls). **g**, Relationship between the number of transcripts that were detected in any given single coacervate and the coacervates respective size. For all boxplots in this figure, the middle bar denotes the median. The lower and upper hinges correspond to the 25th and 75th percentiles. Whiskers extends from the hinge to the largest/smallest value no further than 1.5 x inter-quartile range from the hinge.

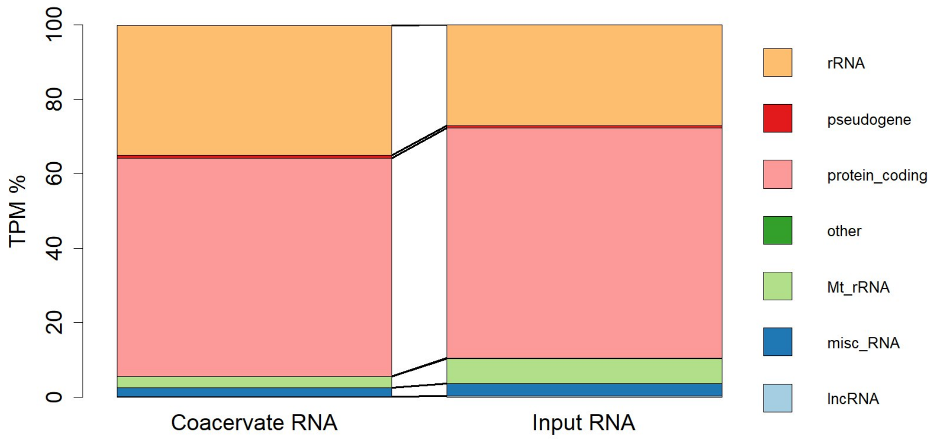

Supplementary Figure 2 | Sequencing of total RNA content from coacervates. Quantitative comparison of RNA biotypes found in 1000 sorted coacervates in comparison to RNA input added to the coacervates before sorting. Libraries for total RNA sequencing were prepared using a random hexamer instead of an oligo-dT primers for reverse transcription.

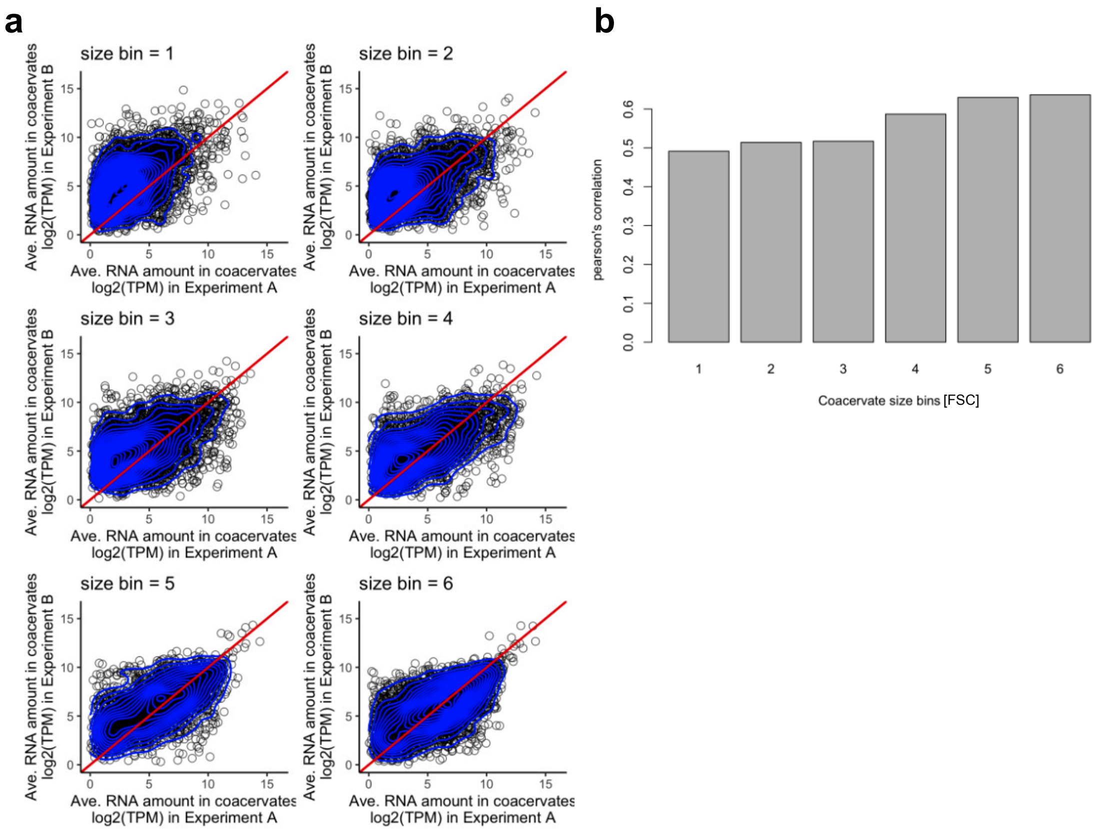

Supplementary Figure 3 | Effect of droplet size on consistency of the number of transcripts found in coacervates. **a**, Experiment-to-experiment variation of the average abundance of each RNA transcript across all CM-Dex:PDDA coacervates in which it was detected as in Fig. 2b. Each plot represents data for a subset of coacervates (size bin) of a given size range. Size bin 1 refers to the smallest and 6 to the largest coacervates. Coacervate size was determined by FACS using the forward scatter (FSC). All size bins are of equal size regarding the number of coacervates they contain. **b**, Comparison of pearson's correlations for all size bins of Supplementary Figure 3a.

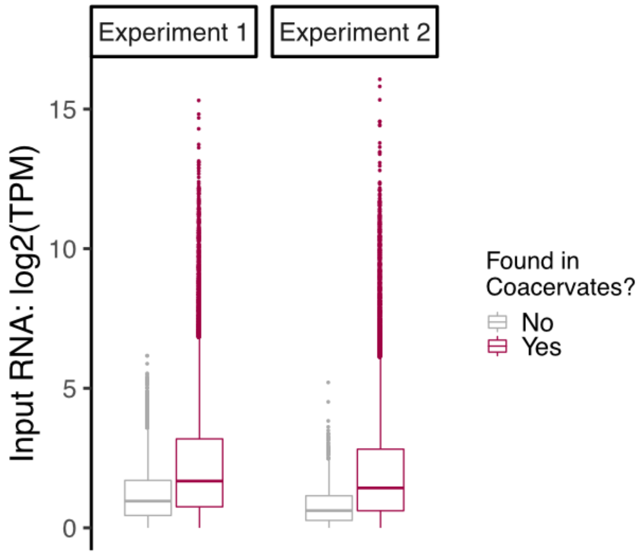

Supplementary Figure 4 | Transcripts not detected in coacervates were not abundant in the input RNA pool. Relationship between transcript abundance in the input of each experiment and whether it was detected in at least one CM-Dex:PDDA coacervate in the respective dataset. Transcript abundance in the input was measured as transcripts per kilobase million (TPM). Experiment 1: n=7141 transcripts not found in coacervates, n=21419 transcripts found in coacervates. Experiment 2: n=2096 transcripts not found in coacervates, n=21978 transcripts found in coacervates. For the boxplots, the middle bar denotes the median. The lower and upper hinges correspond to the 25th and 75th percentiles. Whiskers extends from the hinge to the largest/smallest value no further than 1.5 x inter-quartile range from the hinge.

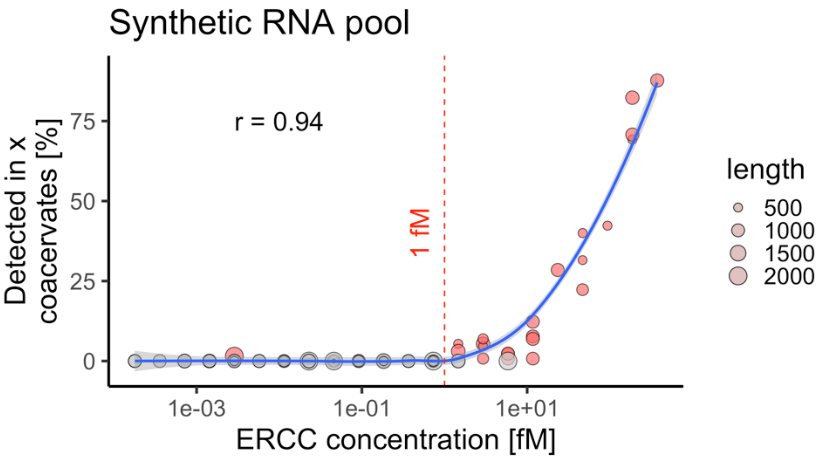

Supplementary Figure 5 | Analysis of synthetic RNA enrichment into coacervates. Synthetic ERCC RNA Spike in mix which consist of 92 transcripts between 250 and 2,000 nucleotides long and correspond to natural eukaryotic mRNAs was analyzed for their enrichment into CM-Dex:PDDA coacervates. Red dots denote ERCC transcripts that were detected in at least one coacervate.

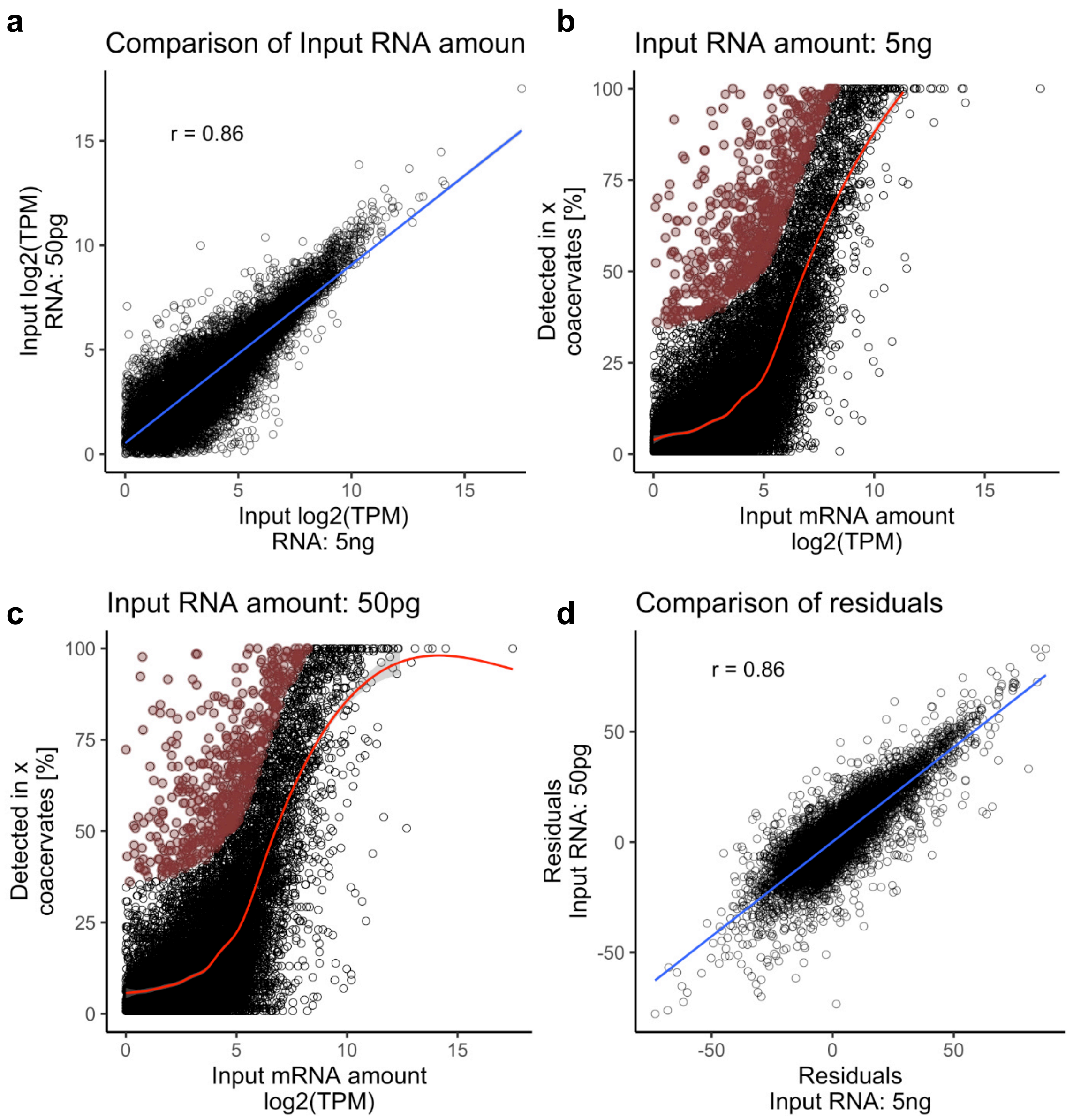

Supplementary Figure 6 | Analysis of input sensitivity. **a**, Comparison of TPM values from input RNA prepared at different concentrations (5ng vs. 50pg). b, Correlation between the frequency of transcript detection and input RNA amount for 5ng of RNA input. Red dots correspond to enriched transcripts (defined as residuals > 30 for generalized additive model). c, Correlation between the frequency of transcript detection and input RNA amount for 50pg of RNA input. Red dots correspond to enriched transcripts (defined as residuals > 30 for generalized additive model). d, Analysis of correlation between enriched (residuals > 30) transcripts obtained from experiments with varying input amounts (5ng vs. 50pg).

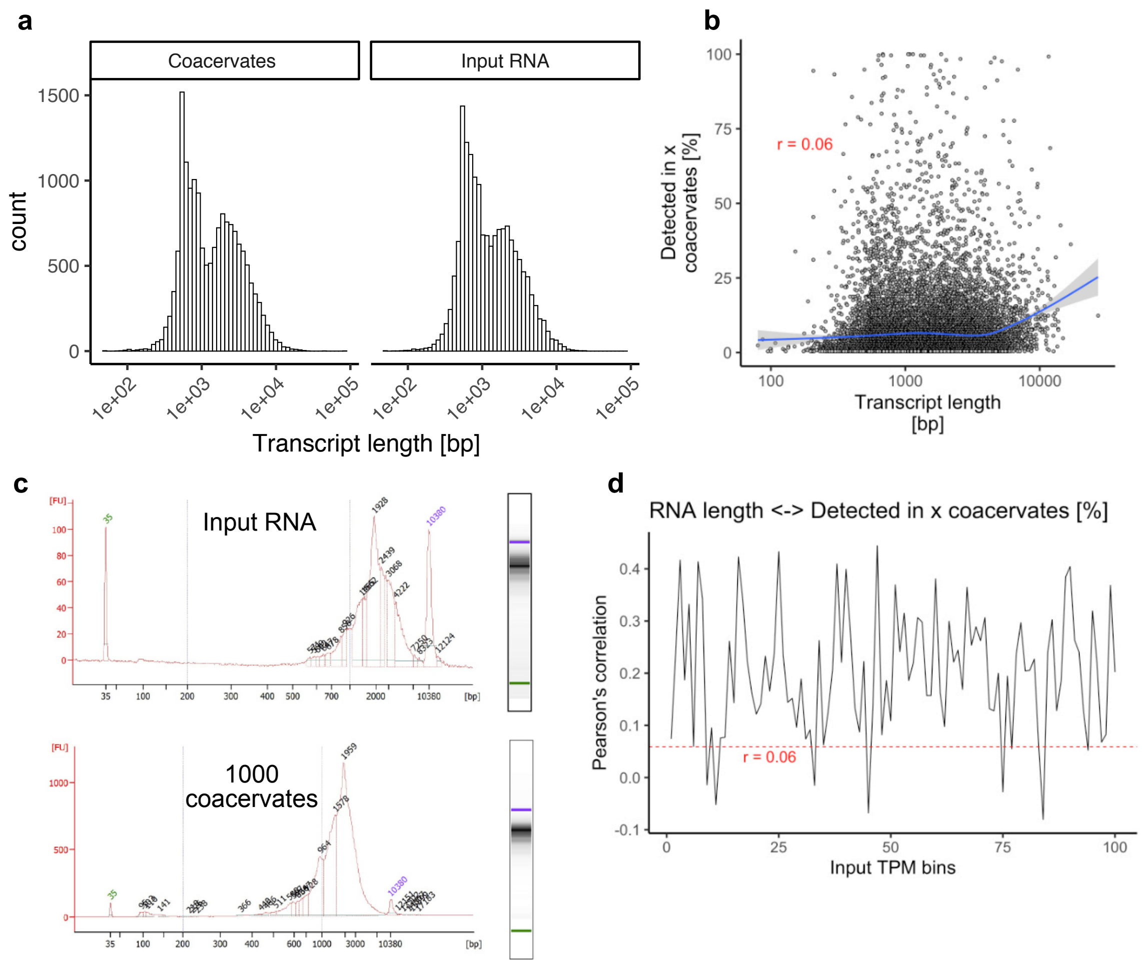

Supplementary Figure 7 | The effect of transcript length on RNA partitioning into coacervates. **a**, Comparison of transcript lengths of all detected RNA transcripts in CM-Dex:PDDA coacervates and the input. **b**, Analysis of frequency of transcript detection in coacervates as a function of transcript length. r = Pearson correlation coefficient. **c**, Bioanalyzer profiles for length comparison of transcripts before ("Input RNA") or after ("1000 coacervates") entering coacervates. **d**, Analysis of the correlation between the frequency of transcript detection in coacervates as a function of transcript length for transcripts with similar abundance in the input ("Input TPM bins"). Red line indicates the correlation between transcript length and their detection frequency when considering all transcripts (compare to Supplementary Figure 7b).

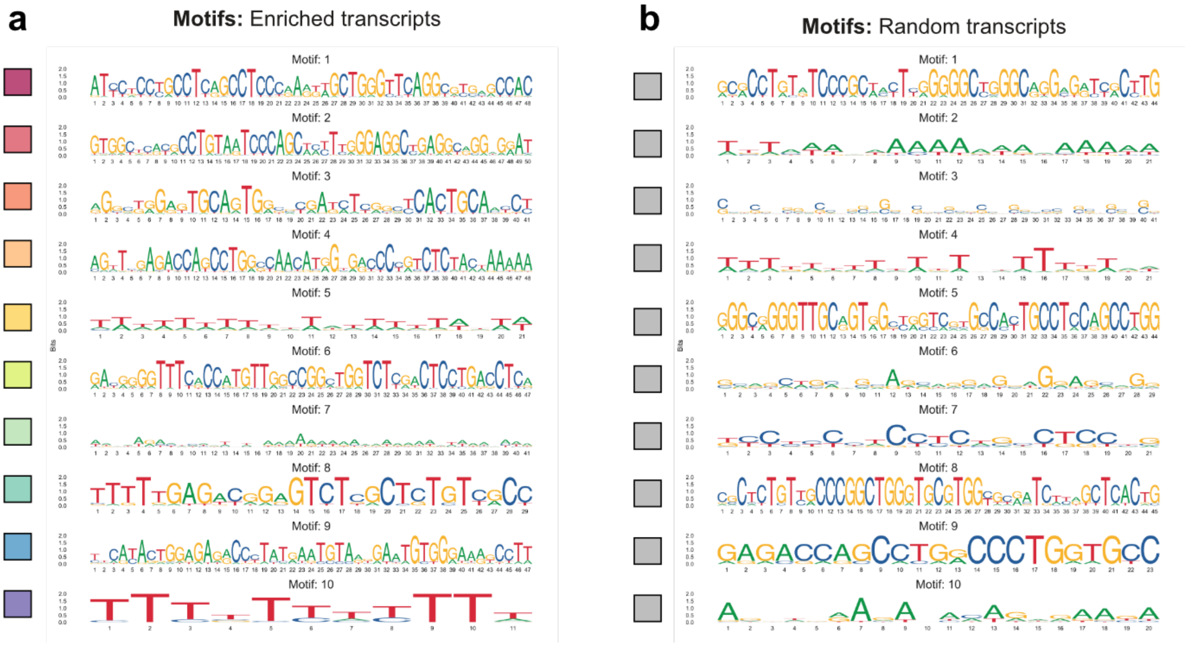

Supplementary Figure 8 | Top motifs in enriched and randomly selected non-enriched transcripts. **a**, Sequences of top 10 motifs (ranked descending from top to bottom) detected in enriched transcripts (as defined in Fig. 3a). **b**, Sequences of top 10 motifs (ranked descending from top to bottom) detected in randomly selected non-enriched transcripts. The number of randomly selected transcripts was equal to the number of enriched transcripts.

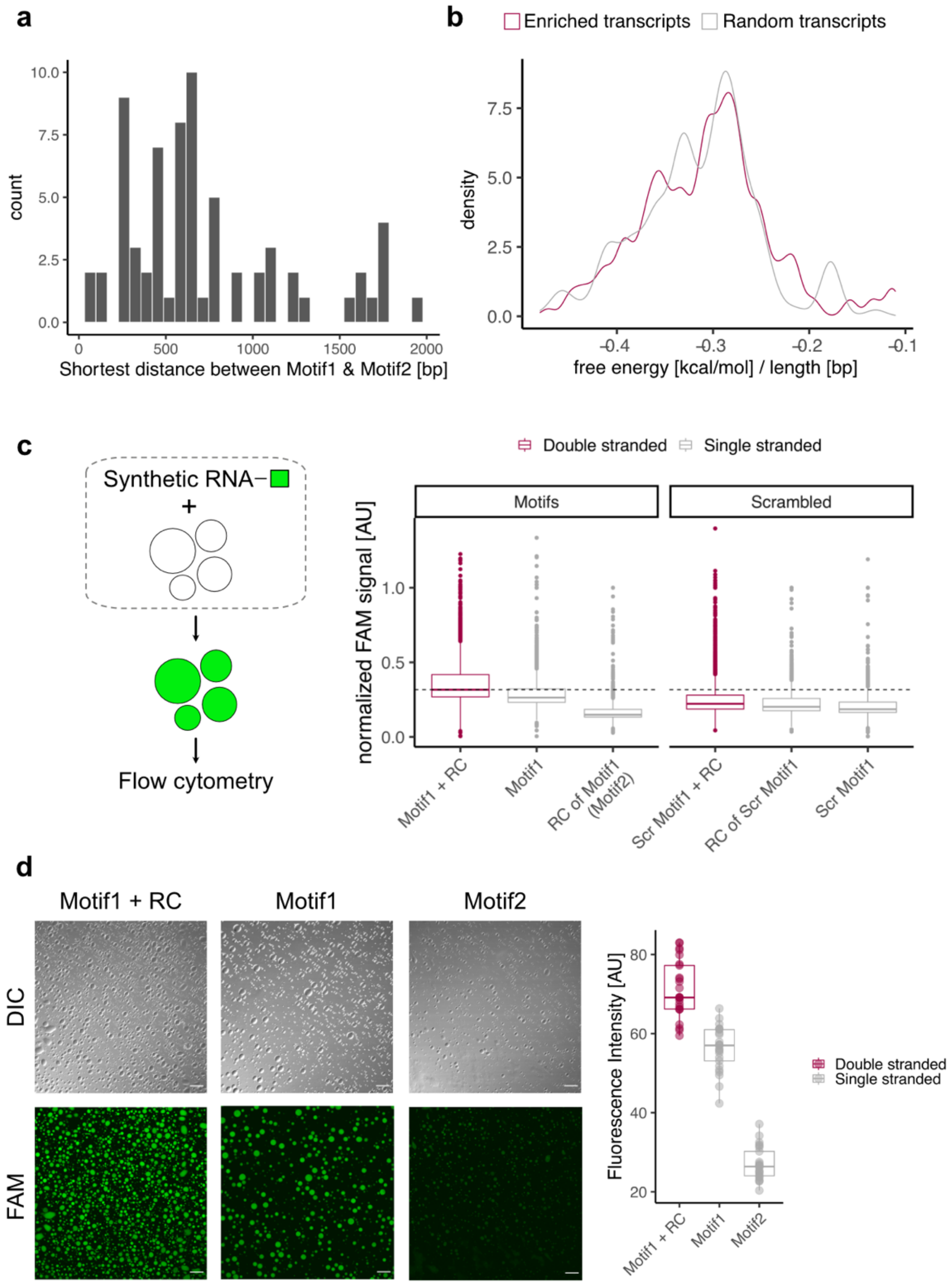
a
count
Shortest distance between Motif1 & Motif2 [bp]
b
Enriched transcripts
Random transcripts
density
free energy [kcal/mol] / length [bp]
c
Synthetic RNA–
+
Flow cytometry
Double stranded
Single stranded
Motifs
Scrambled
normalized FAM signal [AU]
Motif1 + RC
Motif1
RC of Motif1 (Motif2)
Scr Motif1 + RC
RC of Scr Motif1
Scr Motif1
d
Motif1 + RC
Motif1
Motif2
DIC
FAM
Fluorescence Intensity [AU]
Double stranded
Single stranded

Supplementary Figure 9 | Relationship between the two most abundant motifs and RNA complementarity. **a**, Distribution of distances between the two most enriched motifs (Motif1 and Motif2) found among enriched transcripts (see Fig 3a,b). For potential hairpin formation, only the shortest distances between the motif were considered for each transcript. **b**, Comparison of the minimum free energies (normalized for transcript length) of enriched and randomly selected transcripts. **c**, Quantification of CM-Dex:PDDA coacervate uptake of different chemically synthesized RNA sequences. Coacervate uptake of FAM-labelled oligonucleotides were analyzed by flow cytometry. *Motif 1* (most enriched motif - see Fig. 3b and Supplementary Figure 8a), *Motif 2* (its reverse complement (RC)), *Scrambled Motif 1* (scrambled sequence of Motif 1) and the reverse complement (RC) of *Scrambled Motif 1* were analyzed. *Double stranded* refers to pre-mix of Motif 1 or Scrambled Motif 1 with their respective reverse complement (n=10000 coacervates per condition from a single experiment). All comparisons are statistically significant (two-sided t-test, $p < 2e-16$, Bonferroni adjusted). For all boxplots in this figure, the middle bar denotes the median. The lower and upper hinges correspond to the 25th and 75th percentiles. Whiskers extends from the hinge to the largest/smallest value no further than 1.5 x inter-quartile range from the hinge. AU = arbitrary units. **d**, Validation of differential uptake of chemically synthesized RNA sequences by confocal microscopy. Coacervate uptake of FAM-labelled Motif1, Motif2 or both motifs annealed to each other was imaged and quantified. (n=25 coacervates per condition from a single experiment) Scale bar = 50µm. AU = arbitrary units.

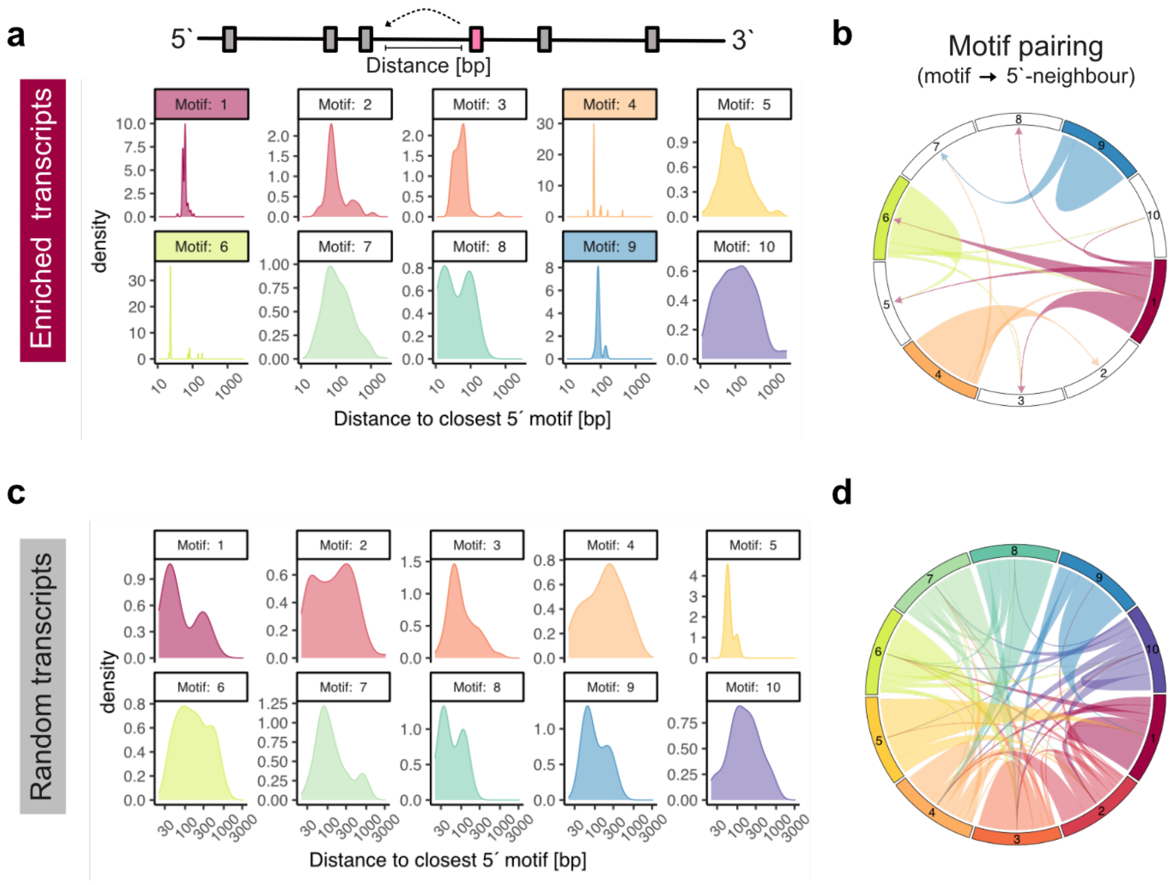

Supplementary Figure 10 | Sequence motif distances on transcripts. **a**, Distribution of distances between each motif (detected in enriched transcripts) and its closest 5`neighbour on a given transcript. Colored facets highlight motifs with narrow distributions. **b**, Circos plot depicting how many times each highlighted motif (see Supplementary Figure 10a) pairs with the other motifs as their respective 5` neighbor. **c**, Distribution of distances between each motif (detected in randomly selected transcripts) and its closest 5`neighbor. **d**, Circos plot depicting how many times each motif pairs with the other motifs as their respective 5` neighbor.

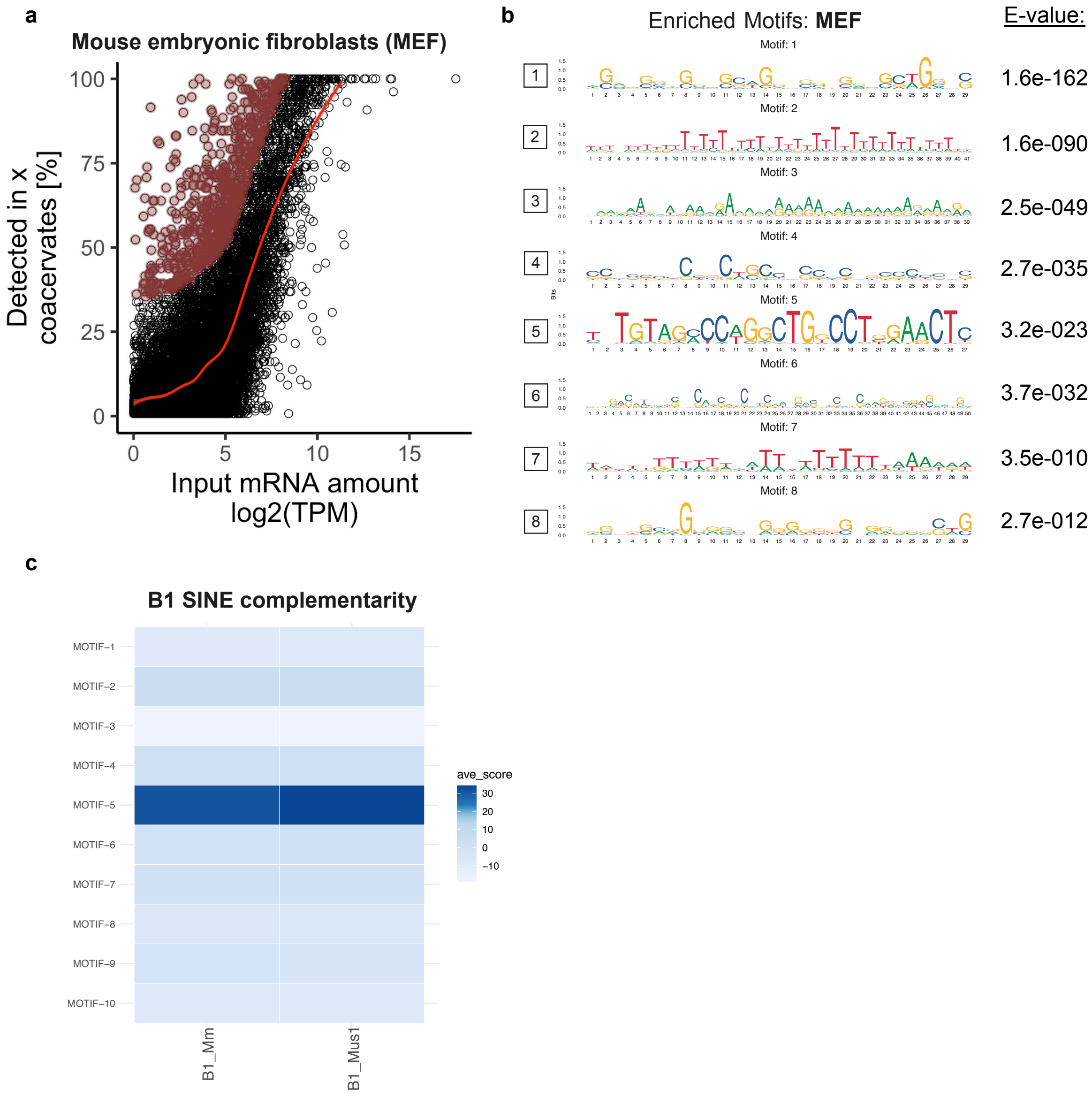

Supplementary Figure 11 | Sequence motif analysis of enriched RNA transcripts derived from murine cells into coacervates. **a**, Uptake of RNA isolated from mouse embryonic fibroblasts into CM-Dex:PDDA coacervates quantified as a function of their abundance in the input pool. Red dots correspond to enriched transcripts defined as residuals > 30 for generalized additive model). **b**, Motif enrichment analysis of enriched RNAs (marked in red in a). Sequences displayed are the eight significantly enriched motifs ranked descending from top to bottom. **c**, MEF motif similarity to B1 Sine elements. Heatmap represents pairwise alignment (Smith-Waterman) of the reverse complement of each motif with B1 SINE sequences (B1_Mm, B1_Mus1) as annotated by RepBase. Color intensity represents alignment score.

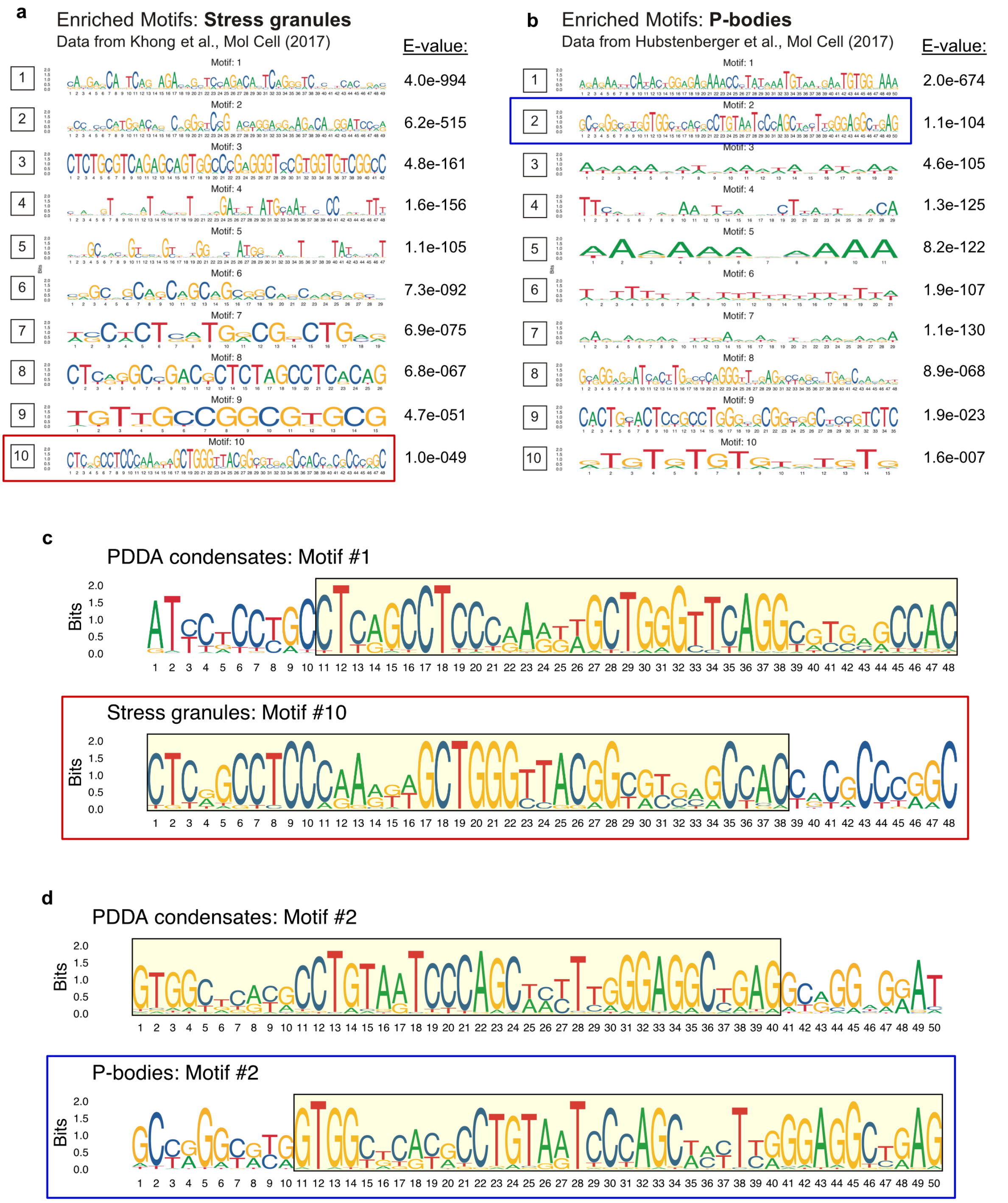

Supplementary Figure 12 | Motif enrichment analysis in stress granules and p-bodies. **a**, Bulk transcriptome data from stress granules isolated from human U2OS cells[15] was analyzed for motif enrichment in 100 protein coding genes most enriched in stress granules. Red rectangle highlights the most enriched motif found in CM-Dex:PDDA coacervate. **b**, Motif enrichment analysis of bulk transcriptome obtained from p-bodies (isolated from human HEK293 cells)[20]. 100 protein coding genes most enriched in p-bodies were analyzed. Blue rectangle highlights second most enriched motif in CM-Dex:PDDA coacervate data. **c**, Comparison of sequence similarity between Motif #1 enriched in PDDA coacervates and Motif #10 enriched in stress granules. **d**, Comparison of sequence similarity between Motif #2 enriched in PDDA coacervates and Motif #2 enriched in P-bodies

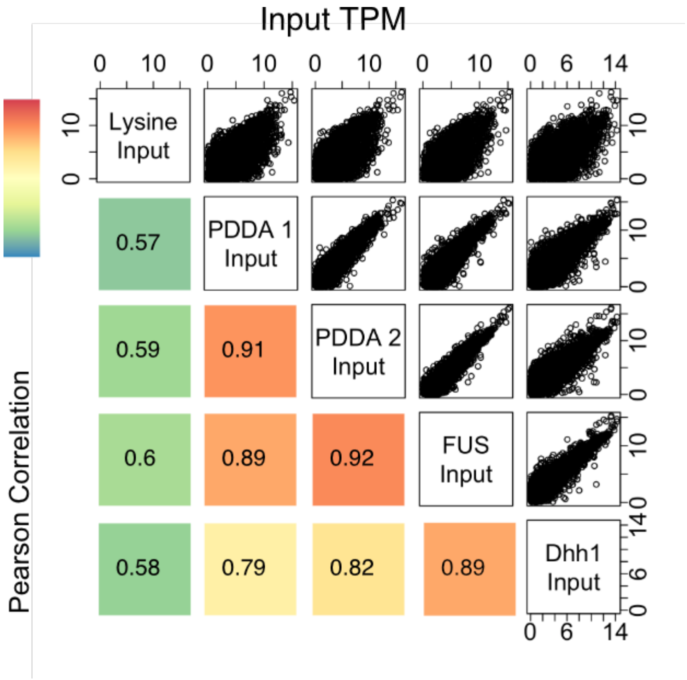

Supplementary Figure 13 | Experiment-to-experiment variation of input RNA abundances. Scatter plots and corresponding Pearson correlations comparing the abundances of all input transcripts (log_2(TPM)) across different experiments and condensate types. Colors represent magnitude of correlation.

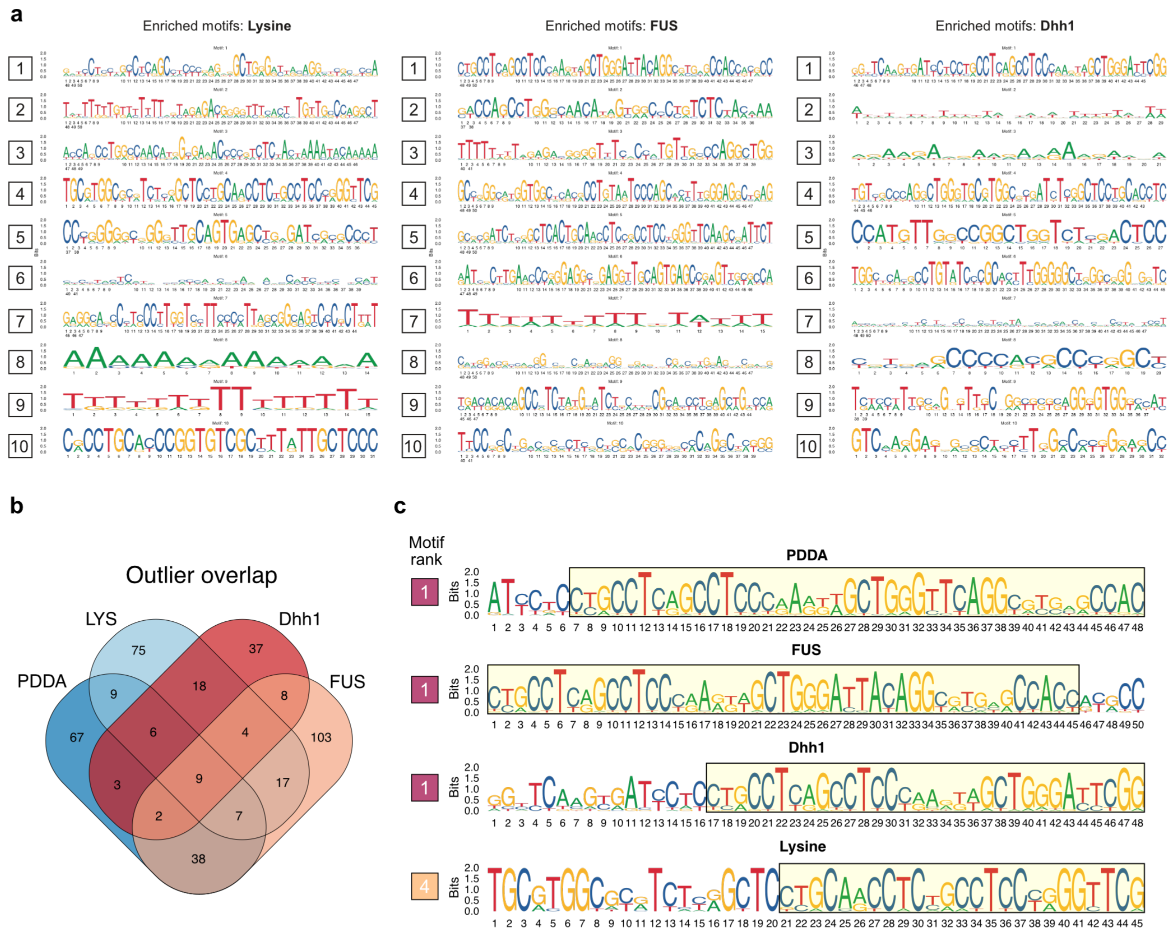

Supplementary Figure 14 | Sequence motif analysis of enriched transcripts across condensate types. **a**, Sequences of top 10 motifs (ranked descending from top to bottom) detected in enriched transcripts of Lysine-CM-Dextran, FUS or Dhh1 droplets. **b**, Quantification of overlap between transcript that are enriched (residuals > 30 - see Fig 3a) in different condensate types. **c**, Comparison of motif similarity between the top motif of the PDDA condensates and sequence similar motifs found in enriched transcripts of each condensate type.

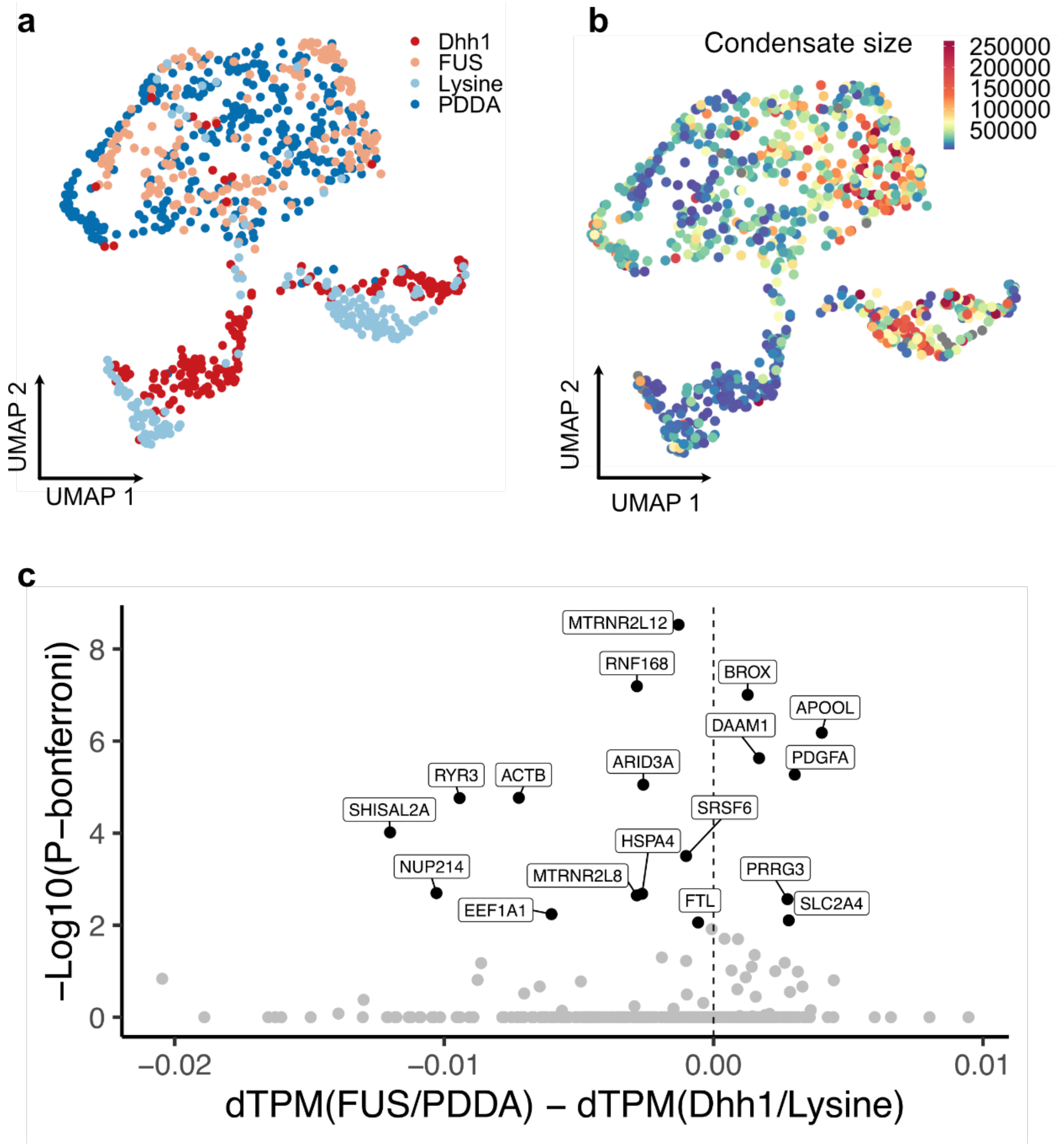

Supplementary Figure 15 | Global comparison of RNA content across all condensate types. **a**, Uniform Manifold Approximation and Projection (UMAP) analysis reduces the dimensionality of the data in order to visualize condensate similarities and differences across thousands of genes. Each dot represents a condensate. Colors represent different condensate types. **b**, Same UMAP as in a with color code representing the size of the condensate as measured by FACS. Legend values correspond to forward-scatter (FSC) values obtained from FACS analysis. **c**, Analysis of differentially expressed gene between the two major clusters FUS/PDDA and Dhh1/Lysine. Significant genes (Bonferroni-adjusted p-value < 0.05) are displayed as black dots labelled with gene names.
