# Supplementary material for: Characterization of RNA content in individual phase-separated coacervate microdroplets
Source: Nat Commun. 2022 May 12;13:2626. doi: 10.1038/s41467-022-30158-1 (PMC9098875; doi:10.1038/s41467-022-30158-1)
Supplement: Supplementary file 3 — Description of Additional Supplementary Files [file 41467_2022_30158_MOESM3_ESM.pdf]

Description of Additional Supplementary Files

File Name: Supplementary Data 1
Description: Transcript abundance per biotype upon random priming reverse transcription.

File Name: Supplementary Data 2
Description: Analysis of differentially expressed gene between the two major clusters FUS/PDDA and Dhh1/Lysine

File Name: Supplementary Data 3
Description: Chemically synthesized motif sequences

File Name: Supplementary Data 4
Description: Amino acid sequence of recombinant proteins
