# Supplementary material for: Characterization of RNA content in individual phase-separated coacervate microdroplets
Source: Nat Commun. 2022 May 12;13:2626. doi: 10.1038/s41467-022-30158-1 (PMC9098875; doi:10.1038/s41467-022-30158-1)
Supplement: Supplementary file 8 — Reporting Summary [file 41467_2022_30158_MOESM8_ESM.pdf]

nature portfolio

Corresponding author(s): Damian Wollny

Last updated by author(s): 22-02-25

Reporting Summary

Nature Portfolio wishes to improve the reproducibility of the work that we publish. This form provides structure for consistency and transparency in reporting. For further information on Nature Portfolio policies, see our Editorial Policies and the Editorial Policy Checklist.

Statistics

For all statistical analyses, confirm that the following items are present in the figure legend, table legend, main text, or Methods section.

n/a	Confirmed	
☐	☒	The exact sample size (*n*) for each experimental group/condition, given as a discrete number and unit of measurement
☒	☐	A statement on whether measurements were taken from distinct samples or whether the same sample was measured repeatedly
☐	☒	The statistical test(s) used AND whether they are one- or two-sided<br>*Only common tests should be described solely by name; describe more complex techniques in the Methods section.*
☒	☐	A description of all covariates tested
☐	☒	A description of any assumptions or corrections, such as tests of normality and adjustment for multiple comparisons
☐	☒	A full description of the statistical parameters including central tendency (e.g. means) or other basic estimates (e.g. regression coefficient) AND variation (e.g. standard deviation) or associated estimates of uncertainty (e.g. confidence intervals)
☐	☒	For null hypothesis testing, the test statistic (e.g. *F*, *t*, *r*) with confidence intervals, effect sizes, degrees of freedom and *P* value noted<br>*Give P values as exact values whenever suitable.*
☒	☐	For Bayesian analysis, information on the choice of priors and Markov chain Monte Carlo settings
☒	☐	For hierarchical and complex designs, identification of the appropriate level for tests and full reporting of outcomes
☐	☒	Estimates of effect sizes (e.g. Cohen's *d*, Pearson's *r*), indicating how they were calculated

Our web collection on statistics for biologists contains articles on many of the points above.

Software and code

Policy information about availability of computer code

Data collection	Illumina high-throughput sequencing, FACSDiva (v8.0.2), FV10-ASW (v04.02.03.06)
Data analysis	R version 3.6.2, Python 3.6.3, MEME Suite (v5.0.5), MAST (v5.0.5), RNAfold (v2.4.12), Kallisto (v0.44.0), STAR (v2.7.9a),

For manuscripts utilizing custom algorithms or software that are central to the research but not yet described in published literature, software must be made available to editors and reviewers. We strongly encourage code deposition in a community repository (e.g. GitHub). See the Nature Portfolio guidelines for submitting code & software for further information.

Data

Policy information about availability of data

All manuscripts must include a data availability statement. This statement should provide the following information, where applicable:

- Accession codes, unique identifiers, or web links for publicly available datasets
- A description of any restrictions on data availability
- For clinical datasets or third party data, please ensure that the statement adheres to our policy

Sequence data is now freely available in ArrayExpress under the accession code E-MTAB-11348

Field-specific reporting

Please select the one below that is the best fit for your research. If you are not sure, read the appropriate sections before making your selection.

☒ Life sciences ☐ Behavioural & social sciences ☐ Ecological, evolutionary & environmental sciences

For a reference copy of the document with all sections, see nature.com/documents/nr-reporting-summary-flat.pdf

Life sciences study design

All studies must disclose on these points even when the disclosure is negative.

Sample size	For determining the number of coacervates to be sequenced, we initially chose numbers (100-200) based on our experience when establishing single-cell sequencing in the lab (e.g. Camp et al., PNAS 2015). Upon coacervate sequencing we adjusted the sample number (300-400 coacervates in particular for CM-Dex:PDDA coacervates since the manuscript is largely based on this type of coacervate) in order to accurately represent the heterogeneity of the population.
Data exclusions	Very few coacervates were discarded based on the quality of the sample. Specifically, we observed that some coacervates showed low number of detected genes despite large size. We observed that these specific coacervates had very low mapping rates indicating poor quality libraries. Hence, we excluded large coacervates with low mapping rates using the following parameters: mapping < 5% for coacervates of size FSC > 2e4. See "Data processing, quality control and analysis" as well as https://github.com/wollnylab/single_coacervate_seq for more details.
Replication	All datasets were obtained in independent experiments. We sequenced 2 datasets (256 coacervates) for CM-Dex:pLys coacervates, 2 datasets (342 coacervates) for CM-Dex:PDDA coacervates, 1 dataset (186 coacervates) for FUS coacervates and 1 dataset (187 coacervates) for Dhh1 coacervates. More information at https://github.com/wollnylab/single_coacervate_seq
Randomization	Coacervate sampling was performed at random, since index-sorting on a FACS machine is inherently random.
Blinding	Investigators were not blinded during sequencing data acquisition. All data analysis upon sequencing were intended to be unbiased in every quantitative comparison that was performed throught the manuscript.

Reporting for specific materials, systems and methods

We require information from authors about some types of materials, experimental systems and methods used in many studies. Here, indicate whether each material, system or method listed is relevant to your study. If you are not sure if a list item applies to your research, read the appropriate section before selecting a response.

Materials & experimental systems

n/a	Involved in the study	
☒	☐	Antibodies
☒	☐	Eukaryotic cell lines
☒	☐	Palaeontology and archaeology
☒	☐	Animals and other organisms
☒	☐	Human research participants
☒	☐	Clinical data
☒	☐	Dual use research of concern

Methods

n/a	Involved in the study	
☒	☐	ChIP-seq
☐	☒	Flow cytometry
☒	☐	MRI-based neuroimaging

Flow Cytometry

Plots

Confirm that:

☒ The axis labels state the marker and fluorochrome used (e.g. CD4-FITC).

☒ The axis scales are clearly visible. Include numbers along axes only for bottom left plot of group (a 'group' is an analysis of identical markers).

☒ All plots are contour plots with outliers or pseudocolor plots.

☒ A numerical value for number of cells or percentage (with statistics) is provided.

Methodology

Sample preparation	See "Single coacervate index sorting"

Instrument	BD FACSAria IIIu
Software	BD FACSDiva and R library "flowCore" for costum analysis. Code is available at https://github.com/wollnylab/single_coacervate_seq
Cell population abundance	Does not apply, since no cells were sorted but synthetic coacervates instead
Gating strategy	Gating strategy is detailed in Suppl. Fig. 1b

☒ Tick this box to confirm that a figure exemplifying the gating strategy is provided in the Supplementary Information.
